# Validation and simplification of the MR PREDICTS @24H model for outcome prediction after endovascular thrombectomy for acute ischemic stroke

**DOI:** 10.1177/17474930261430342

**Published:** 2026-02-23

**Authors:** Xi Li, Bob Roozenbeek, Theo van Walsum, Daniel Bos, H Bart van der Worp, Bart J Emmer, Charles BL Majoie, Robert J van Oostenbrugge, Diederik WJ Dippel, Hester F Lingsma, The ICAI Stroke Lab

**Affiliations:** 1 Department of Public Health, Erasmus MC, University Medical Center Rotterdam, Rotterdam, The Netherlands; 2 Department of Neurology, Erasmus MC, University Medical Center Rotterdam, Rotterdam, The Netherlands; 3 Department of Radiology and Nuclear Medicine, Erasmus MC, University Medical Center Rotterdam, Rotterdam, The Netherlands; 4 Department of Epidemiology, Erasmus MC, University Medical Center Rotterdam, Rotterdam, The Netherlands; 5 Department of Neurology and Neurosurgery, UMC Utrecht Brain Center, University Medical Center Utrecht, Utrecht, The Netherlands; 6 Department of Radiology and Nuclear Medicine, Amsterdam University Medical Centers, University of Amsterdam, Amsterdam, The Netherlands; 7 Department of Neurology, Cardiovascular Research Institute Maastricht (CARIM), Maastricht University Medical Centre, Maastricht, The Netherlands

**Keywords:** Prediction model, ischemic stroke, endovascular therapy, prognosis, model validation, model simplification, post-treatment outcome

## Abstract

**Background:**

Outcome prediction after endovascular thrombectomy (EVT) for ischemic stroke is important for patient counseling and rehabilitation planning. MR PREDICTS @24H, a nine-predictor model, showed excellent performance in predicting functional outcome at 90 days of patients with acute ischemic stroke. With the expanding treatment indications, we validated the model for patients receiving EVT within 24 hours after stroke onset and simplified it for easier clinical implementation.

**Methods:**

We used individual patient data from the Dutch MR CLEAN Registry (2014–2018), a prospective observational cohort enrolling patients treated with EVT, and three randomized controlled trials MR CLEAN-MED, MR CLEAN-NOIV, and MR CLEAN-LATE (2018–2022). We included patients with an intracranial large-vessel occlusion in the anterior circulation treated with EVT within 24 hours of symptom onset or last seen well. We assessed the effect of predictors on functional outcome (modified Rankin Scale [mRS]) at 90 days with ordinal logistic regression. Predicted probabilities of functional independence (mRS 0–2) and survival (mRS 0–5) were derived from the model formula. We evaluated predictive performance with discrimination (C statistic) and calibration (intercept, slope). The model was simplified by excluding predictors based on the Akaike information criterion (AIC). We applied leave-one-study-out cross-validation to evaluate heterogeneity in model performance between the cohorts.

**Results:**

The validation cohort included 6154 patients: 4737 from the Registry and 1417 from the trials. External validation of the original model showed excellent discrimination in predicting functional independence (C statistic 0.91, 95% CI 0.90–0.92) and survival (C statistic 0.90, 95% CI 0.89–0.91). The simplified model, comprising four predictors—NIHSS at 24 hours after EVT, age, pre-stroke mRS, and symptomatic intracranial hemorrhage—performed comparably (functional independence C statistic 0.91, 95% CI 0.90–0.92; and survival 0.89, 95% CI 0.88–0.90). Cross-validation revealed heterogeneity between LATE and the other cohorts, with the model overestimating the probability of functional independence in LATE (observed 39.1% vs predicted 44.2%), whereas the observed and predicted probability of survival was similar (75.5% vs 75.7%).

**Conclusions:**

A simplified version of MR PREDICTS @24H including only four predictors performed as good as the full model, providing a practical tool that can be applied one day after EVT for reliable outcome estimation. Further validation and updating of the model are needed for patients treated in the late time window (6–24 h).

## Graphical abstract

**Graphical abstract:**
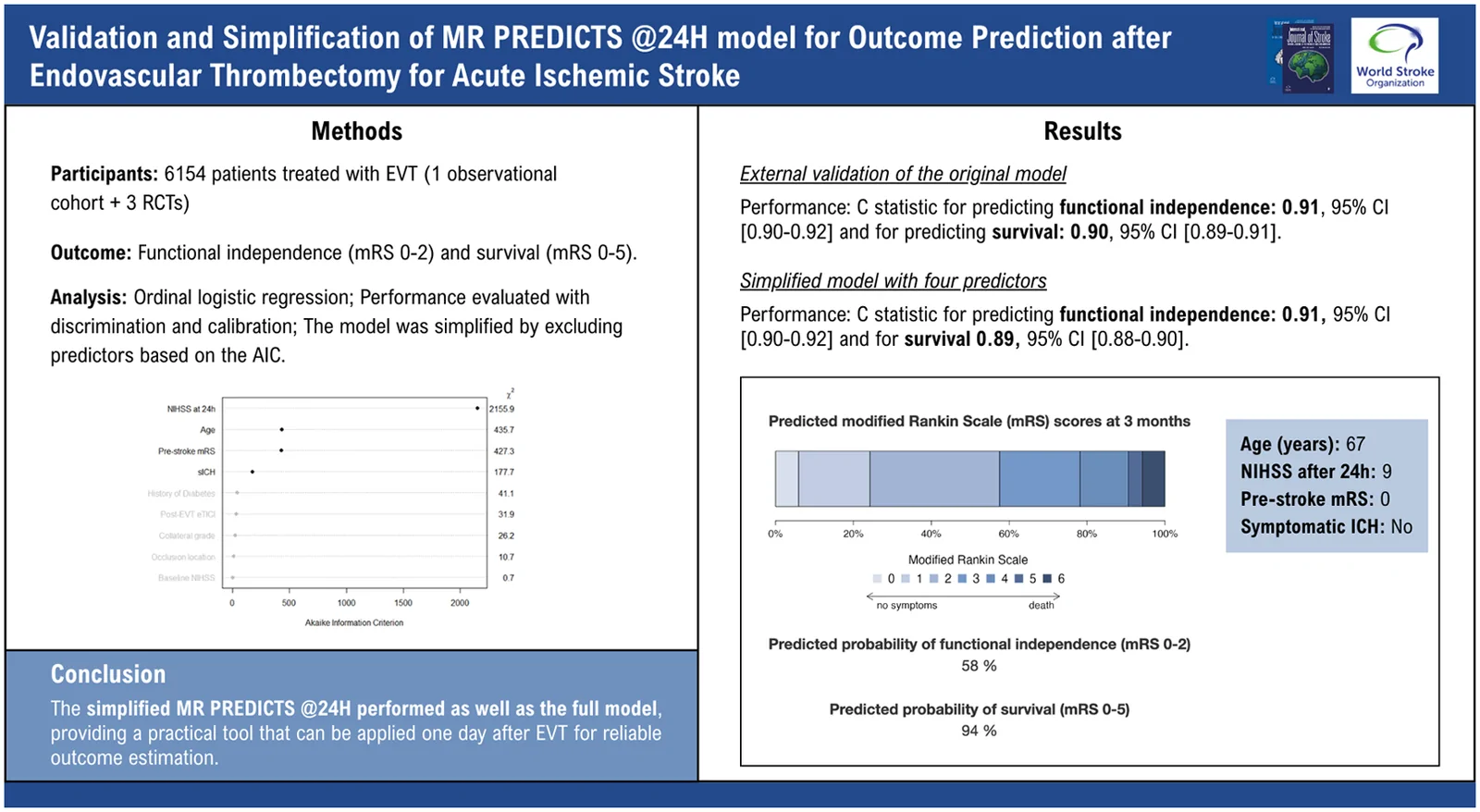

## Introduction

Endovascular thrombectomy (EVT) has been proven as an effective treatment for acute ischemic stroke caused by intracranial proximal arterial occlusions.^1^ As it continues to be studied and applied for broader indications, there is greater variability in patient characteristics and outcomes after treatment. Prediction models developed from large datasets have demonstrated superior accuracy in prediction than physicians, especially when multiple factors need to be considered simultaneously.^2^ These models can be valuable tools for providing accurate prediction for patient counseling and decision-making for rehabilitation plans.

The MR PREDICTS @24H prediction model predicts functional outcomes at 90 days for individual stroke patients treated with EVT and includes nine predictors on pre- and post-procedural clinical and imaging characteristics.^3^ The study highlighted the added value of post-procedural characteristics, including NIHSS at 24 hours, reperfusion grade, and symptomatic intracranial hemorrhage (sICH), in outcome prediction. The model showed excellent discriminative ability when externally validated in an observational study of patients treated with EVT within 12 hours from symptom onset or last seen well. With advancements in EVT techniques, devices, as well as the expanding treatment indications, especially for patients treated in the late time window (6–24 hours),^4,5^ the model requires validation and update for contemporary patient populations. Given that the nine predictors in the model may not always be readily available in clinical practice, a simplified version using fewer and easily accessible variables could enhance clinical usability without compromising predictive accuracy.

In this study, we externally validated and updated the MR PREDICTS @24H model. In addition, we investigated whether the model can be simplified to fewer predictors while maintaining comparable performance to enhance clinical usability.

## Methods

### External validation cohort

We combined patient data from the MR CLEAN Registry and three RCTs (MR CLEAN-MED, MR CLEAN-NOIV, and MR CLEAN-LATE) to externally validate and update the MR PREDICTS @24H model.

MR CLEAN Registry^6^ is a prospective, nationwide, observational study in 18 centers that provided EVT in the Netherlands. All consecutive patients undergoing EVT for acute ischemic stroke from March 2014 until December 2018 were registered. There were no restrictions on upper age limit, minimum ASPECTS, or collateral score in the inclusion criteria. The central medical ethics committee of the Erasmus MC, Rotterdam, the Netherlands, granted permission to carry out the study as a registry (MEC-2014-235), for which no written consent was necessary.

The three multicenter RCTs within the Dutch Collaboration for New Treatments of Acute Stroke (CONTRAST) consortium included acute ischemic stroke patients with intracranial large-vessel occlusion (LVO) in the anterior circulation. MR CLEAN-MED^7^ evaluated the benefit of periprocedural heparin and/or aspirin along with EVT initiated within 6 hours of symptom onset; MR CLEAN-NOIV^8^ compared intravenous alteplase prior to EVT vs EVT alone within 4.5 hours after symptom onset; MR CLEAN-LATE^9^ assessed the effect of EVT, compared to best medical treatment, for patients in the late time window (6–24 hours after symptom onset or last seen well (LSW)). All participants provided written informed consent, and each RCT was approved by the medical ethics committee of Erasmus MC (MEC-2017-366, MEC-2017-367, MEC-2017-368).

For this analysis, we included patients aged 18 years or older, with an occlusion of the intracranial carotid artery (ICA, ICA-T) or middle (M1/M2) or anterior (A1/A2) cerebral artery, demonstrated by CT angiography (CTA), MR angiography (MRA), or Digital subtraction angiography (DSA), and who had undergone arterial puncture within 24 hours after symptom onset.

### MR PREDICTS @24H prediction model

MR PREDICTS @24H^3^ was previously developed using individual patient data pooled from seven RCTs in the HERMES collaboration. These RCTs, conducted between 2010 and 2014, compared the efficacy of EVT with standard care in adult patients with ischemic stroke caused by LVO in the anterior circulation. The model was externally validated previously in part of the MR CLEAN registry, which included all patients treated with EVT from March 2014 to November 2017. The model included nine predictors: age, baseline NIHSS score, pre-stroke mRS, history of diabetes, occlusion location, collateral score, reperfusion grade (modified Treatment in Cerebral Infarction [mTICI] score), 24-hour NIHSS, and sICH at 24 hours after EVT.

### Outcome of interest

The primary outcome was functional status at 90 days, evaluated with the modified Rankin Scale (mRS).^10^ The analysis treated mRS as a full ordinal scale in the ordinal logistic regression model, and we subsequently derived the probabilities of functional independence (mRS 0–2) and survival (mRS 0–5) from the regression model.

### Statistical analysis

#### Validation of the full model

We assessed the effect of the predictors on the outcome (mRS) with a multivariable ordinal logistic regression model. Definitions of the predictors were used as described in the MR PREDICTS @24H paper.^3^ Non-linear terms (i.e. age and 24-hour NIHSS score) were kept the same as in the original model with restricted cubic spline functions with three knots. Patients with missing outcomes were included for imputation but excluded from the final analyses. Missing values were imputed with multiple imputations (m = 5). Results of the analyses were pooled with Rubin’s rules.^11^ External validation was performed by applying the original model formula to the new dataset. Probabilities for functional independence (mRS 0–2) and survival (mRS 0–5) were derived from the formula reported in the original paper using the intercept for observational data.^3^

Model performance was evaluated with discrimination and calibration. Discrimination refers to the model’s capability to differentiate between patients with favorable and unfavorable outcomes, assessed with the concordance statistic (C statistic).^12^ We calculated Harrell’s C statistic for the ordinal mRS, as well as for the predicted probabilities of functional independence (mRS 0–2 vs mRS 3–6) and survival (mRS 0–5 vs mRS 6). Calibration measures how well the predicted probabilities align with the observed outcomes. We evaluated calibration graphically by plotting the observed proportion of patients with the outcome against the predicted probabilities calculated from the model. Calibration was quantified using the calibration intercept and slope. The intercept reflects whether the model’s predictions systematically overestimate or underestimate outcomes in the validation cohort, and ideally it should be 0. The calibration slope ideally should be 1, indicating that the predicted probabilities perfectly align with the observed outcomes.^12^

#### Recalibration for the full model

We updated the intercept of the original full model to correct for “calibration in the large,” which accounts for differences in baseline risk between the development and validation cohorts, such that the average predicted probability matched the observed event rate in the validation population.

#### Model simplification

To simplify the model, we used analysis of variance (ANOVA) plot to rank predictors based on their contribution to the net improvement in model fit, quantified by the increase in the Chi-square statistic while adjusting for degrees of freedom. The most important predictors were selected based on the plot, and their corresponding Akaike information criterion (AIC) values were used to determine a post hoc threshold. This AIC threshold was then applied as a stopping rule for backward stepwise selection, repeated across 200 bootstrap samples to assess the stability and robustness of the modeling strategy.

We assessed the internal validity of the final simplified model with 200 bootstrap resampling to report the optimism-corrected model performance and estimated a uniform shrinkage factor. We multiplied the regression coefficients by the shrinkage factor and re-estimated the model intercept. To assess heterogeneity in model performance across cohorts, we applied leave-one-study-out cross-validation. Specifically, each study was iteratively excluded from the development set. The model was then developed using data from the remaining studies and validated on the one left out, ensuring that each study served as an independent validation cohort once.

Patients with missing outcomes were used for data imputation but excluded from the validation cohort for analyses. This study followed the TRIPOD + AI (Transparent Reporting of Clinical Prediction Models that use Regression or Machine Learning Methods for Individual Prognosis or Diagnosis) reporting guidelines.^13^ All statistical analyses were performed with R version 4.3.2, with the following packages: mice, rms, Hmisc, dplyr, PredictionTools.

### Sensitivity analysis

We evaluated the predictive value of time from symptom onset or LSW to groin puncture based on the ANOVA plot and compared the model’s performance with and without including it.

We also examined whether adding pre-stroke mRS and sICH provided incremental value beyond the two strongest predictors, age and 24-hour NIHSS. As AUC is often insensitive to incremental improvements in prediction, we used net reclassification index (NRI)^14^ to quantify how many patients were correctly reclassified into risk categories for functional independence and survival when comparing the four- versus two-predictor models.

## Results

After excluding 456 patients from the MR CLEAN Registry due to missing mRS scores, we included 6154 patients in the validation cohort: 4737 from MR CLEAN Registry, 628 from MR CLEAN-MED, 536 from MR CLEAN-NOIV, 253 from the EVT arm of MR CLEAN-LATE. Compared to the derivation cohort (the HERMES dataset), patients in the validation cohort were older (age 72 [63–81] vs. 67 [57–76]), had more often pre-stroke disability (pre-stroke mRS ⩾ 2: 19.6% vs. 4.6%), fewer had good collaterals (baseline collateral grade 3: 21% vs 40%), and more often achieved a post-eTICI score of 3 (33.3% vs 8.5%). Fewer patients achieved functional independence (43% vs 48%), and the survival rate is lower (73% vs 86%) at 3 months (Table 1, Supplemental Table S1).

**Table 1.** Patient characteristics in the derivation and validation cohorts.

	Derivation cohort (HERMES) n = 781	Validation cohort (Registry + 3 RCTs) n = 6154	Absolute standardized mean difference
Characteristic			
Age, median (IQR), y	67 (57, 76)	72 (63, 81)	0.36
Sex			
Men	414 (53)	3219 (52)	0.02
Women	367 (47)	2935 (48)	0.02
NIHSS score at baseline, median (IQR)	17 (14, 21)	15 (10, 19)	0.33
Systolic blood pressure, median (IQR), mm Hg	144 (130, 159)	150 (132, 167)	0.25
Serum glucose level, median (IQR), mmol/L	6.7 (5.9, 7.8)	6.8 (5.9, 8.1)	0.07
Previous stroke	89/777 (11)	1098/6118 (18)	0.20
Hypertension	426/779 (55)	3180/6067 (52)	0.06
Atrial fibrillation	217/640 (34)	1393/6093 (23)	0.25
Diabetes	120/780 (15)	1012/6126 (17)	0.05
Pre-stroke mRS score			
0	501/605 (83)	3900/6033 (65)	0.42
1	76/605 (13)	949/6033 (16)	0.09
2	19/605 (3.1)	519/6033 (9)	0.25
⩾3	9/605 (1.5)	665/6033 (11)	0.40
Occlusion location			
ICA-(T)	198/733 (27)	1455/5954 (24)	0.07
M1	473/733 (65)	3294/5954 (55)	0.21
M2 or other	62/733 (8.5)	1205/5954 (20)	0.33
ASPECTS scale, median (IQR)	8 (7, 9)	9 (8, 10)	0.50
Collateral score			
0	5/602 (0.8)	319/5867 (5)	0.25
1	81/602 (14)	1997/5867 (34)	0.48
2	268/602 (45)	2337/5867 (40)	0.10
3	248/602 (41)	1214/5867 (21)	0.44
Treatment with intravenous alteplase	678/781 (87)	4027/6146 (66)	0.51
Time from stroke onset to arterial puncture, median (IQR), min	240 (185, 299)	190 (140, 269)	0.53
General anesthesia	227/776 (29)	1167/5842 (20)	0.21
Duration of the procedure, median (IQR), min	64 (40, 91)	54 (35, 80)	0.28
Outcome measures			
Reperfusion grade (mTICI/eTICI)^a^			
0	54/715 (7.6)	854/5851 (15)	0.24
1	19/715 (2.7)	133/5851 (2)	0.05
2A	98/715 (14)	935/5851 (16)	0.06
2B	483/715 (68)	1319/5851 (23)	NA
2C	NA	662/5851 (11)	NA
3	61/715 (8.5)	1948/5851 (33)	0.63
NIHSS score at 24 h, median (IQR)	9 (4, 16)	8 (3, 16)	0.08
sICH at 24 h	28/770 (3.6)	405/6154 (6.6)	0.14
mRS score at 3 mo, median (IQR)	3 (1, 4)	3 (2, 6)	0.38
mRS 0–2 at 3 mo	371 (48)	2633 (43)	0.10
Survival at 3 mo	671 (86)	4514 (73)	0.33

ASPECTS: Alberta Stroke Program Early CT Score; HERMES: Highly Effective Reperfusion Evaluated in Multiple Endovascular Stroke Trials; ICA(-T): intracranial carotid artery (terminus); M1: middle cerebral artery segment 1; M2: middle cerebral artery segment 2; mRS: modified Rankin Scale; mTICI: modified treatment in cerebral infarction; eTICI: expanded treatment in cerebral infarction; NA: not applicable; NIHSS: National Institutes of Health Stroke Scale; sICH: symptomatic intracranial hemorrhage. Categorical values are presented as No. (%) and continuous values as median (IQR). Absolute standardized mean differences (SMD) quantify the magnitude of between-cohort differences, with values >0.10 indicating non-negligible difference.

a Reperfusion was scored with mTICI in the development cohort, and eTICI in the validation cohort.

The effect of predictors on ordinal mRS in the validation cohort was similar to that in the derivation cohort (HERMES dataset) (Table 2). For patients in the three RCTs, the effect of baseline NIHSS, M2 occlusion, and post-eTICI score on the ordinal mRS became non-significant (Supplemental Table S2).

**Table 2.** Main effects of the predictors in the derivation and validation cohorts.

	Derivation cohort (HERMES)	Validation cohort (Registry + 3 RCTs)
	n = 781	n = 6154
	Adjusted common OR (95% CI)	
Age, per year^a^		
<65 years	0.99 (0.98–1.01)	1.01 (1.00–1.02)
⩾65 years	0.94 (0.91–0.96)	0.94 (0.93–0.94)
Baseline NIHSS, per point	1.03 (1.00–1.06)	1.00 (0.99–1.00)
Diabetes mellitus	0.48 (0.33–0.71)	0.66 (0.57–0.75)
Pre-stroke mRS, per point	0.62 (0.46–0.84)	0.61 (0.58–0.64)
Occlusion location		
ICA(-T)	1.0 (reference)	1.0 (reference)
M1	1.29 (0.94–1.75)	1.13 (1.01–1.28)
M2 or other	2.03 (1.19–3.48)	1.28 (1.09–1.49)
Collateral score, per point	1.25 (0.94–1.64)	1.19 (1.12–1.27)
Post-procedural reperfusion grade (eTICI), per point	1.19 (1.02–1.40)	1.09 (1.06–1.12)
24-hour NIHSS, per point^a^		
<12 points	0.71 (0.68–0.75)	0.80 (0.79–0.82)
⩾12 points	0.86 (0.83–0.90)	0.86 (0.84–0.87)
sICH	0.28 (0.10–0.76)	0.17 (0.13–0.23)

ASPECTS: Alberta Stroke Program Early CT Score; ICA(-T): intracranial carotid artery(-terminus); IV: intravenous; M1: middle cerebral artery segment 1; M2: middle cerebral artery segment 2; mRS: modified Rankin Scale; eTICI: expanded treatment in cerebral infarction; NA: not applicable; NIHSS: National Institutes of Health Stroke Scale; sICH: symptomatic intracranial hemorrhage. The association between predictors and functional outcome was assessed with ordinal logistic regression modeling. Adjusted common odds ratios with 95% CI were results from multivariable regression analysis and reflect the effect on the reversed mRS (an OR > 1 corresponds with better functional outcome).

a Included in the model as a non-linear term with a restricted cubic spline with three knots.

### External validation of the original full model

**Figure 1.**
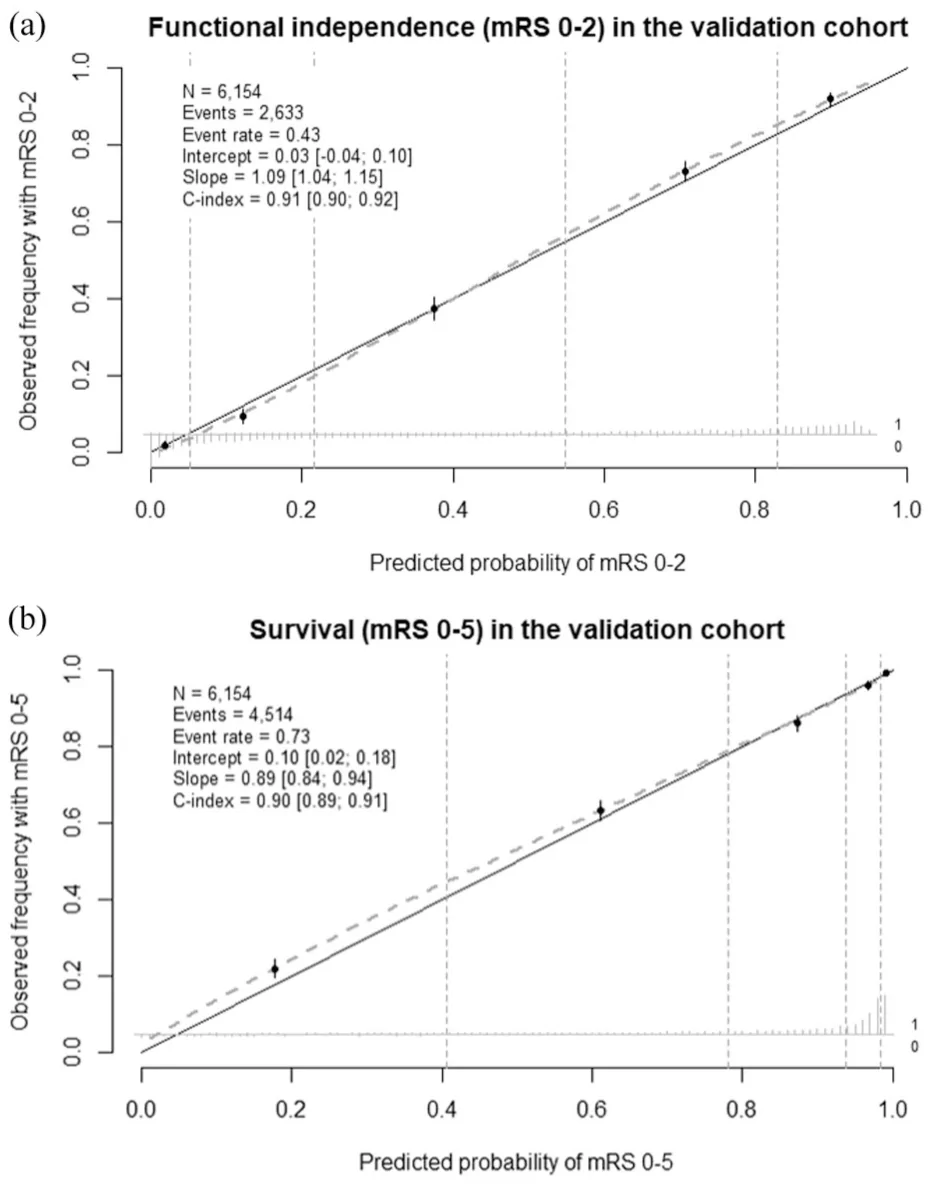
Calibration plot of validation of the original MR PREDICTS @24H model. (a) Predictive performance of functional independence (modified Rankin Scale [mRS] 0–2) in the validation cohort. The overall observed proportion of patients with mRS 0–2 in the validation cohort was similar to the predicted proportion with our model (43% vs 42%). The linear bar chart shows the distribution of patients with (= 1) and without (= 0) the observed outcome. (b) Predictive performance of survival (modified Rankin Scale [mRS] 0–5) in the validation cohort. The overall observed proportion of patients with mRS 0–5 in the validation cohort was similar to the predicted proportion with our model (73% vs 72%). The linear bar chart shows the distribution of patients with (= 1) and without (= 0) the observed outcome.

The original MR PREDICTS @24H model showed good discrimination in predicting ordinal mRS (C statistic = 0.83 [95% CI 0.83–0.84]) and excellent discrimination for functional independence (C statistic = 0.91 [95% CI 0.90–0.92]) and survival (C statistic = 0.90 [95% CI 0.89–0.91]) (Table 3). The calibration plot showed good agreement between the predicted probability and observed frequency for functional independence (intercept = 0.03, slope = 1.09) and survival (intercept = 0.10, slope = 0.89) (Figure 1). The mean observed probability of functional independence was 43.0%, compared to a predicted mean of 42.4%. The observed probability of survival was 73.0% versus a predicted mean of 72.4%. The model was recalibrated by updating the intercept for each mRS level (Supplemental Appendix).

**Table 3.** Model performance measures (with 95% CI) in derivation and validation cohorts.

Measure	Derivation cohort (HERMES)	Previous validation cohort (part of Registry)	Current validation cohorts (Registry + 3 RCTs)
External validation of the original model			
C statistic			
Ordinal mRS	0.84 (0.84 to 0.85)	0.84 (0.83 to 0.84)	0.83 (0.83 to 0.84)
Functional independence (mRS 0–2)	0.92 (0.91 to 0.92)	0.91 (0.90 to 0.92)	0.91 (0.90 to 0.92)
Survival (mRS 0–5)	0.87 (0.85 to 0.88)	0.89 (0.88 to 0.90)	0.90 (0.89 to 0.91)
Calibration intercept			
Functional independence (mRS 0–2)	NA	0.61 (0.50 to 0.74)	0.03 (–0.04 to 0.10)
Survival (mRS 0–5)	NA	−0.25 (−0.37 to −0.13)	0.10 (0.02 to 0.18)
Calibration slope			
Functional independence (mRS 0–2)	NA	0.98 (0.92 to 1.05)	1.09 (1.04 to 1.15)
Survival (mRS 0–5)	NA	0.86 (0.80 to 0.94)	0.89 (0.84 to 0.94)
Internal validation of the simplified model (corrected for optimism)			
C statistic			
Ordinal mRS	NA	NA	0.83 (0.82 to 0.84)
Functional independence (mRS 0–2)	NA	NA	0.91 (0.90 to 0.92)
Survival (mRS 0–5)	NA	NA	0.89 (0.88 to 0.90)

mRS: modified Rankin Scale; NA: not applicable.

### Model simplification

The ANOVA plot identified NIHSS at 24 hours, age, pre-stroke mRS, and sICH as the four predictors that contributed most to explaining the variability in mRS at 90 days (Supplemental Figure S1), while others showed limited predictive value. A post hoc AIC threshold of 150 was used to repeat this predictor-selection procedure in bootstrap resampling. In 199 out of 200 bootstrap resampling, the same four predictors were selected, while one sample included a fifth predictor, history of diabetes mellitus.

### Predictive performance of the simplified model

The internally validated C statistic of the simplified model, corrected for optimism, was 0.83 (95% CI 0.82–0.84) for ordinal mRS, 0.91 (95% CI 0.90–0.92) for functional independence, and 0.89 (95% CI 0.88–0.90) for survival. (Table 3)

**Figure 2.**
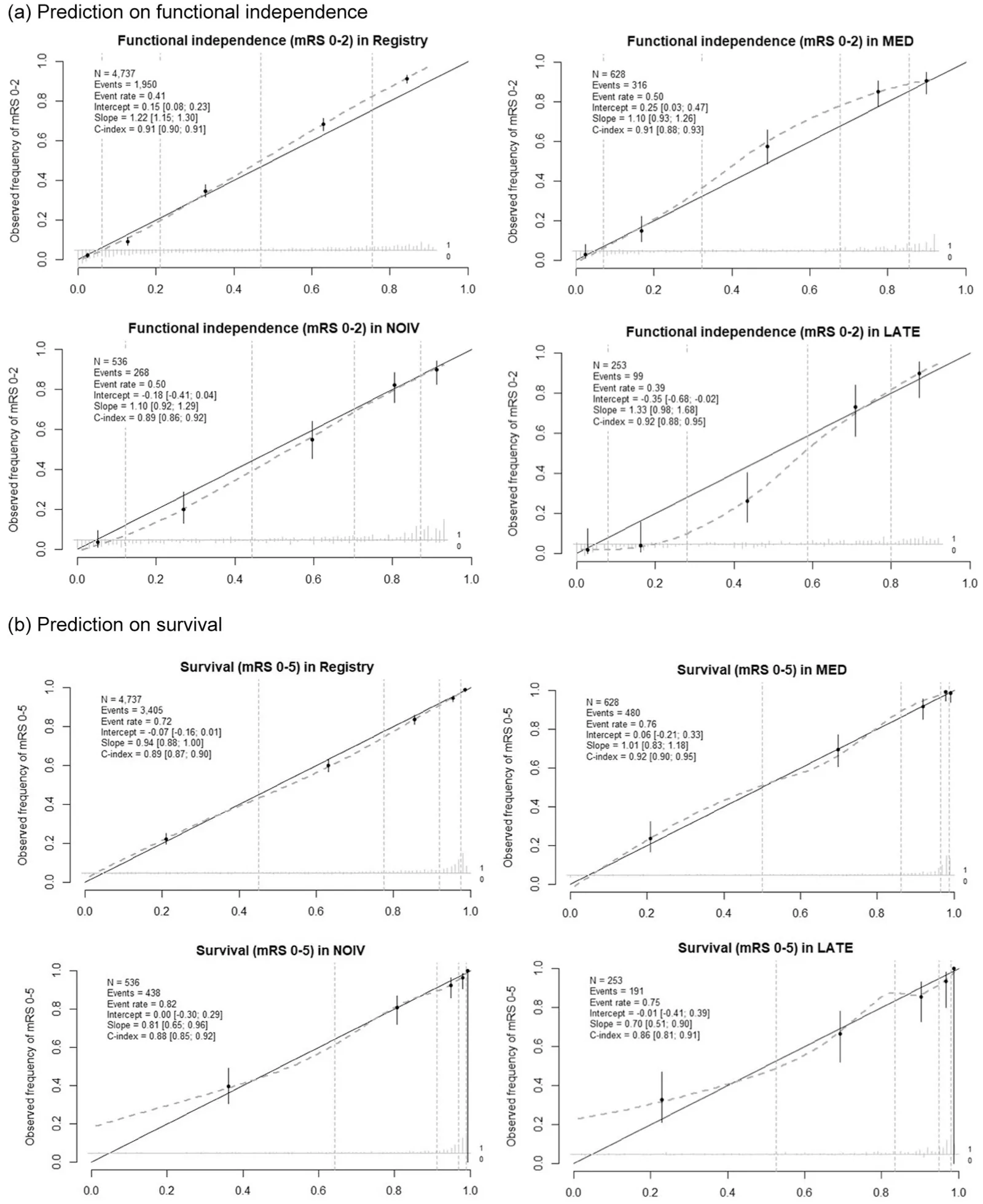
Calibration plot from leave-one-study-out cross-validation of the simplified MR PREDICTS @24H model. (a) Prediction on functional independence. (b) Prediction on survival. The simplified model was fitted using data from three of the four datasets and validated on the left-out dataset. The figure shows the calibration performance of the model for predicting functional independence (a) and survival (b) in the validation cohort.

In leave-one-study-out cross-validation, overall, the model showed good discrimination and calibration across different cohorts (Figure 2). When the model was developed using data from the other three studies and validated in LATE, the plot showed an overestimation of the probability of functional independence (calibration intercept = –0.35, slope = 1.33), especially for patients with a low chance of functional independence. The mean observed and predicted probability was 39.1% versus 44.2%. For prediction of survival in LATE, the predicted probabilities were on average too extreme (calibration intercept = –0.01, slope = 0.70), particularly too low for the low-risk group. The mean observed and predicted probability was similar (75.5% vs 75.7%).

The regression equations of both full and simplified model are available in the Supplemental Material. The online tool is accessible for clinical use at www.mrpredicts.com.

### Sensitivity analysis

The ANOVA plot showed limited predictive value of onset-to-groin time (AIC = 0.8) compared to the other predictors, with no improvement in model performance when added.

Reducing the model to only two predictors, age and NIHSS at 24 h, resulted in a small but statistically significant decrease in C statistic for functional independence (ΔC statistic = –0.02, p < 0.001) and survival (ΔC statistic = –0.01, p < 0.001). Moreover, the four-predictor model improved risk classification, with an NRI of 0.58 for functional independence and 0.62 for survival, indicating more accurate prediction for 58% and 62% of patients compared to the two-predictor model (Supplemental Table S3).

## Discussion

The original MR PREDICTS @24H model showed excellent discrimination and calibration for predicting functional independence and survival of patients with ischemic stroke caused by a proximal occlusion in the anterior circulation and treated with EVT. To ease clinical usability, we simplified the model to only four predictors—NIHSS at 24 h, age, pre-stroke mRS, and sICH—while maintaining comparable predictive performance.

Three external validation studies performed outside the Netherlands demonstrated excellent predictive performance and generalizability of MR PREDICTS @24H across countries and treatment time windows.^15–17^ Our validation results confirmed the model’s predictive performance in a contemporary population with a broader selection of patients.

With that, we aimed to simplify it for clinical usability while maintaining its performance. As the original model had undergone variable selection based on clinical knowledge and significance testing with multivariable analysis, repeating the selection process would yield the same set of predictors. Therefore, we visualized the relative contribution of these predictors to the model using an ANOVA plot (based on AIC). The post hoc AIC threshold was selected purely to replicate the selection process and does not carry intrinsic clinical value. While the exact AIC value may not be reproducible in other settings, this approach provided a pragmatic way to simplify the model while testing the stability of the modeling strategy with bootstrapping. The ANOVA plot showed that post-EVT clinical variables contributed significantly more to outcome prediction, surpassing baseline clinical and imaging variables. Notably, 24-hour NIHSS was the most influential predictor. Prior studies have identified it as the strongest predictor for 90-day functional recovery over baseline and early change in NIHSS.^18^ It captures both initial stroke severity and the time-dependent treatment effect, which may explain why baseline NIHSS and onset-to-groin time variables ranked lowest in the ANOVA plot. It also outperformed eTICI score, likely because recanalization does not fully reflect final reperfusion, whereas post-EVT NIHSS better distinguishes between clinically meaningful and futile reperfusion.^19^

Leave-one-study-out cross-validation revealed discrepancies between cohorts. Despite that, the overall discrimination ability remains strong across cohorts. In the MR CLEAN-MED trial, a higher incidence of sICH was observed due to the periprocedural use of antithrombotic drugs. The model was able to effectively account for this as sICH is a significant predictor of functional outcomes. Miscalibration was observed in the MR CLEAN LATE trial when the model was trained using data from the other three cohorts. The model overestimated the probability of functional independence for patients in the low-risk quartile. This does not necessarily indicate poor model performance but rather reflects differences between cohorts. Patients in LATE were selected based on the sole presence of collaterals and treated in the late time window. They had lower ASPECTS, lower intravenous thrombolysis rates, and lower successful recanalization rates, which are features typical of late-window patients.^20,21^

Adding time from symptom onset or LSW to groin puncture and witnessed stroke onset did not improve predictive performance, likely because the 24-hour NIHSS already captures much of the time effect. In addition, in LATE, nearly 85% of patients had unwitnessed stroke onset, making time estimates unreliable and limiting their predictive value. Importantly, the external validation study in a U.S.-based cohort^15^ demonstrated excellent discrimination and calibration of the model in late-window patients (beyond 12 hours), supporting its generalizability. Combined with the excellent discrimination observed in our study, the model is likely to perform well in late-treated patients. Nevertheless, dedicated external validation of the simplified model with a larger dataset of this subgroup remains necessary.

A recently developed post-EVT prediction score, HERMES-24,^22^ with only age and 24-hour NIHSS, performed as good as the MR PREDICTS @24H, raising discussion about whether 24-hour NIHSS itself is sufficient to predict the outcome. With the reclassification metrics, we found that with four predictors, the model correctly reclassified a substantial number of patients compared to the model with two predictors. Given that the two additional predictors are easily accessible in clinical practice, the four-predictor model offers a practical and more reliable tool for clinical decision-making.

### Strengths and limitations

A key strength of this study is the heterogeneity of the validation cohort. It included patients treated with EVT in real-world clinical practice, those receiving periprocedural antithrombotic drugs or intravenous thrombolytics along with EVT, and those treated in the late time window (6–24 hours). This makes the model applicable to a wide range of ischemic stroke patients for outcome prediction after EVT. In addition, the full model was updated to a more contemporary population, ensuring its relevance to the current clinical practice. Predictors for the simplified model were derived from the set of variables that have already been validated in the original model, which leverages their established predictive value and reduces the risk of omitting key factors. Physicians can choose between the models based on available data at the time of outcome consultation. With the rapid development of prediction models, including those using machine learning methods, our model with only four predictors that are commonly used and easily accessible one day after EVT can achieve excellent predictive performance. Its simplicity ensures easy clinical usability, reproducibility, and facilitates future validation in diverse settings.

There are some limitations in this study. First, the number of patients treated in the late time window is relatively small, which may lead to potential under-representation of this group in the validation cohort. This may explain the observed miscalibration and the relatively wide confidence interval of the C statistic. However, the C statistic suggested excellent discrimination in late-treated patients. Further validation and update of the model are needed to ensure its robustness in this subgroup. Second, given the small number of patients with ASPECTS < 5, the model should be interpreted with caution, and further validation in larger observational datasets with more large-core patients will be necessary. Finally, ischemic stroke patients with occlusion in the distal medium vessel or posterior circulation were not included in the dataset; whether the model is generalizable to these patients require further research.

## Conclusion

MR PREDICTS @24H model demonstrated excellent discrimination when externally validated in a contemporary and heterogeneous cohort. A simplified version of the model with only four predictors demonstrated similar performance as the full model, offering a more practical tool for reliable outcome estimations and individualized rehabilitation planning. Further update and validation of the model are needed for patients treated in the late time window (6–24 hours).

## Supplemental Material

sj-pdf-1-wso-10.1177_17474930261430342 – Supplemental material for Validation and simplification of the MR PREDICTS @24H model for outcome prediction after endovascular thrombectomy for acute ischemic stroke Supplemental material, sj-pdf-1-wso-10.1177_17474930261430342 for Validation and simplification of the MR PREDICTS @24H model for outcome prediction after endovascular thrombectomy for acute ischemic stroke by Xi Li, Bob Roozenbeek, Theo van Walsum, Daniel Bos, H Bart van der Worp, Bart J Emmer, Charles BL Majoie, Robert J van Oostenbrugge, Diederik WJ Dippel and Hester F Lingsma in International Journal of Stroke
