# Supplementary material for: Validation and simplification of the MR PREDICTS @24H model for outcome prediction after endovascular thrombectomy for acute ischemic stroke
Source: Int J Stroke. 2026 Feb 23;21(8):1119–29. doi: 10.1177/17474930261430342 (PMC13601691; doi:10.1177/17474930261430342)
Supplement: Supplementary file 1 [file sj-pdf-1-wso-10.1177_17474930261430342.pdf]

Supplement materials

Appendix:

R code: Equation of full model with new intercepts after recalibration

R code: Equation of simplified model

Table S1 Patient characteristics in each of the validation cohorts

Table S2 Effects of the predictors in each of the validation cohorts

Table S3 Reclassification of patients comparing the model with four predictors versus two predictors

Figure S1: Predictor selection for model simplification

Appendix
R code (Updated full model equation)[a]

```
plogis((+0.00907192*age - 4.83914e-05*pmax(age-50.5,0)^3 + 0.000118985*pmax(age-
72,0)^3 7.05932e-05*pmax(age-86.5,0)^3 - 0.00333532*NIHSS baseline -
0.563654*diabetes mellitus - 0.508099*pre-mRS + 0.154629*(occlusion location=="M1") +
0.282501*(occlusion location=="M2") + 0.115803*collateral score + 0.0793556*eTICI -
0.252124*NIHSS24h + 0.000148483*pmax(NIHSS24h-1,0)^3 -
0.000272219*pmax(NIHSS24h-11,0)^3 + 0.000123736*pmax(NIHSS24h-23,0)^3 -
1.68195*sICH) + intercept[b])
```

[b] Intercept per each modified Rankin Scale cut-off point after recalibration

mRS cut-off point	New intercept after recalibration
mRS 0 vs 1-6	-1.49033
mRS 0-1 vs 2-6	0.18923
mRS 0-2 vs 3-6	1.73141
mRS 0-3 vs 4-6	2.79131
mRS 0-4 vs 5-6	3.85119
mRS 0-5 vs 6	4.41193

R code (Simplified model equation)[a]

```
plogis((+0.0081076048*Age - 4.8989509e-05*pmax(Age-52,0)^3 +
0.00011897452*pmax(Age-72,0)^3 - 6.9985013e-05*pmax(Age-86,0)^3 -
0.47947152*pre-mRS - 0.239872*NIHSS24h + 0.00017364022*pmax(NIHSS24h-1,0)^3 -
0.00028049575*pmax(NIHSS24h-9,0)^3 + 0.00010685552*pmax(NIHSS24h-22,0)^3 -
1.7072962*sICH) + intercept[c])
```

[c] Intercept per each modified Rankin Scale cut-off point for the simplified model (after uniform shrinkage factor applied)

mRS cut-off point	Intercept after shrinkage
mRS 0 vs 1-6	-1.07411
mRS 0-1 vs 2-6	0.55387
mRS 0-2 vs 3-6	1.99603

mRS 0-3 vs 4-6	2.97187
mRS 0-4 vs 5-6	3.95794
mRS 0-5 vs 6	4.48467

[a] Variable definitions

Variable	Definition
Age	Numeric; in years
NIHSS baseline	Numeric; range 1-42
Diabetes mellitus	Binary; 0=no, 1=yes
Pre-mRS	Numeric; 0=0, 1=1, 2=2, >2=3
Occlusion location	Categorical; ICA-(T), M1, M2
Collateral score	Numeric; range 0-3
eTICI	Numeric; 0=0, 1=1, 2A=2, 2B=3, 2C=4, 3=5
NIHSS 24h	Numeric; range 0-42
sICH	Binary; 0=no, 1=yes

Table S1: Patient characteristics in each dataset of the validation cohort

	Registry N=4737	MED N=628	NOIV N=536	LATE N=253
Characteristic				
Age, median (IQR), y	72 (63, 81)	73 (65, 81)	71 (62, 79)	74 (64, 80)
Sex, male	2477 (52.3)	332 (52.9)	303 (56.5)	107 (42.3)
NIHSS score at baseline, median (IQR)	15 (11, 19)	15 (9, 19)	16 (10, 20)	10 (6, 17)
Systolic blood pressure, median (IQR), mm Hg	150 (132, 167)	150 (134, 166)	150 (133, 169)	155 (135, 175)
Serum glucose level, median (IQR), mmol/L	6.8 (5.9, 8.2)	6.85 (6.0, 8.0)	6.7 (5.9, 7.9)	6.8 (6.0, 8.3)
Previous stroke	842 (17.9)	115 (18.3)	91 (17.0)	50 (19.8)
Hypertension	2491 (53.5)	289 (46.1)	259 (48.4)	141 (56.0)
Atrial fibrillation	1128 (24.1)	156 (24.8)	58 (10.8)	51 (20.2)
Diabetes	793 (16.8)	94 (15.0)	90 (16.8)	35 (13.9)
Prestroke mRS score				
0	2964 (64.2)	420 (67.1)	372 (69.5)	144 (57.1)
1	671 (14.5)	114 (18.2)	99 (18.5)	65 (25.8)
2	372 (8.1)	60 (9.6)	49 (9.2)	38 (15.1)
>=3	613 (13.3)	32 (5.2)	15 (2.8)	5 (2.0)
Occlusion location				
ICA-(T)	1135 (25.0)	165 (26.4)	118 (22.0)	37 (14.6)
M1	2523 (55.6)	308 (49.3)	328 (61.3)	135 (53.4)
M2 or other	883 (19.5)	152 (24.4)	89 (16.5)	81 (32.0)
ASPECTS scale, median (IQR)	9 (8, 10)	9 (8, 10)	9 (8, 10)	9 (7, 10)
Collateral score				
0	238(5.3)	38 (6.1)	32 (6.1)	11 (4.4)
1	1602 (35.8)	176 (28.4)	152 (28.9)	67 (26.9)
2	1713 (38.3)	292 (47.1)	225 (42.8)	107 (43.0)
3	919 (20.6)	114 (18.4)	117 (22.2)	64 (25.7)
Treatment with IV alteplase	3285 (69.5)	466 (74.2)	265 (49.4)	11 (4.3)
Time from stroke onset to arterial puncture, median (IQR), min	193 (146, 265)	175 (144, 228)	133 (105, 180)	735 (578, 935)
General anesthesia	1027 (22.9)	48 (7.9)	73 (14.5)	19 (7.7)
Duration of the procedure, median (IQR), min	55 (35, 81)	47 (30, 70)	47 (31, 70)	55 (32, 75)
Outcome measures				
Reperfusion grade (mTICI)				
0	741 (16.3)	57 (9.8)	36 (7.5)	20 (8.4)
1	115(2.5)	11 (1.9)	6 (1.3)	1 (0.4)
2A	798 (17.5)	47 (8.0)	49 (10.3)	41 (17.2)
2B	1042 (22.9)	120 (20.5)	108 (22.6)	49 (20.5)
2C	479 (10.5)	74 (12.7)	59 (12.3)	50 (20.9)
3	1375 (30.2)	275 (47.1)	220 (46.0)	78 (32.6)

NIHSS score at 24 h, median (IQR)	9 (3, 17)	6 (2, 14)	6 (1, 13)	7 (3, 16)
sICH at 24 h	292(6.2)	66 (10.5)	30 (5.6)	17 (6.7)
mRS at 3m, median(IQR)	3 (2, 6)	2 (1, 5)	2.5 (2, 5)	3 (2, 5)
mRS 0-2 at 3m	1950 (41)	316 (50)	268 (50)	99 (39)
survival at 3m	3405 (72)	480 (76)	438 (82)	191 (75)

Categorical values are presented as No. (%) and continuous values as median (IQR). Abbreviations: ASPECTS, Alberta Stroke Program Early CT Score; HERMES, Highly Effective Reperfusion Evaluated in Multiple Endovascular Stroke Trials; ICA(-T), intracranial carotid artery (terminus); M1, middle cerebral artery segment 1; M2, middle cerebral artery segment 2; mRS, modified Rankin Scale; eTICI, expanded treatment in cerebral infarction; NIHSS, National Institutes of Health Stroke Scale; sICH, symptomatic intracranial hemorrhage.

Table S2: Main effects of the predictors in each dataset of the validation cohort

	Registry	MED	NOIV	LATE
	Adjusted common OR (95% CI)			
Age, per year				
<65 years	1.01 (1.00-1.02)	1.04 (1.01-1.07)	1.00 (0.98-1.03)	1.01 (0.97-1.05)
≥65 years	0.93 (0.92-0.94)	0.95 (0.93-0.97)	0.95 (0.93-0.97)	0.96 (0.92-0.99)
Baseline NIHSS, per point	0.99 (0.98-1.00)	1.00 (0.97-1.03)	1.01 (0.98-1.04)	0.96 (0.91-1.00)
Diabetes mellitus	0.66 (0.56-0.77)	0.53 (0.34-0.82)	0.57 (0.36-0.91)	0.97 (0.47-2.02)
Pre-stroke mRS, per point	0.62 (0.59-0.65)	0.47 (0.38-0.57)	0.61 (0.49-0.77)	0.48 (0.34-0.67)
Occlusion location				
ICA(-T) (reference)				
M1	1.10 (0.97-1.26)	1.28 (0.88-1.86)	1.40 (0.94-2.10)	1.82 (0.88-3.76)
M2 or other	1.29 (1.08-1.54)	1.33 (0.85-2.10)	1.52 (0.87-2.66)	1.51 (0.66-3.47)
Collateral score, per point	1.17 (1.09-1.25)	1.34 (1.11-1.63)	1.46 (1.18-1.80)	0.87 (0.64-1.18)
Post-procedural reperfusion grade (mTICI), per point	1.11 (1.07-1.15)	0.99 (0.90-1.09)	1.11 (0.98-1.25)	1.04 (0.88-1.24)
24h NIHSS, per point				
<12 points	0.81 (0.79-0.82)	0.82 (0.78-0.86)	0.80 (0.76-0.84)	0.76 (0.70-0.82)
≥12 points	0.86 (0.84-0.88)	0.87 (0.83-0.91)	0.90 (0.85-0.94)	0.94 (0.89-0.99)
sICH	0.18 (0.12-0.25)	0.06 (0.03-0.14)	0.29 (0.11-0.75)	0.40 (0.13-1.29)

The association between predictors and functional outcome was assessed with ordinal logistic regression modelling. Adjusted common odds ratios with 95% CI were results from multivariable regression analysis and reflect the effect on the reversed mRS (an OR >1 corresponds with better functional outcome). Abbreviations: ASPECTS, Alberta Stroke Program Early CT Score; ICA(-T), intracranial carotid artery(-terminus); IV, intravenous; M1, middle cerebral artery segment 1; M2, middle cerebral artery segment 2; mRS, modified Rankin Scale; mTICI, modified treatment in cerebral infarction; NA, not applicable; NIHSS, National Institutes of Health Stroke Scale; sICH, symptomatic intracranial hemorrhage.

Table S3: Reclassification of patients comparing the model with four predictors versus two predictors

	Functional independence (mRS 0-2)	Survival (mRS 0-5)
Patients with events		
Increase in predicted probability	81%	78%
Decrease in predicted probability	19%	22%
Patients with non-events		
Decrease in predicted probability	48%	53%
Increase in predicted probability	52%	47%
Mean change in probability		
Increase for events (sensitivity)	2.2%	1.2%
Decrease for non-events (specificity)	1.6%	3.5%

Note: The table shows the percentage of patients who were reclassified when the model extended from two predictors (24h NIHSS+Age) to four predictors (24h NIHSS+Age+Pre-mRS+sICH). Reclassification is shown separately for patients with and without events (functional independence and survival). Correct classification is indicated by an increased predicted probability for patients with events and a decreased predicted probability for patients without events. The bottom row shows the net change in sensitivity and specificity.

Figure S1 Predictor selection for simplified model based on Akaike information criterion (AIC)

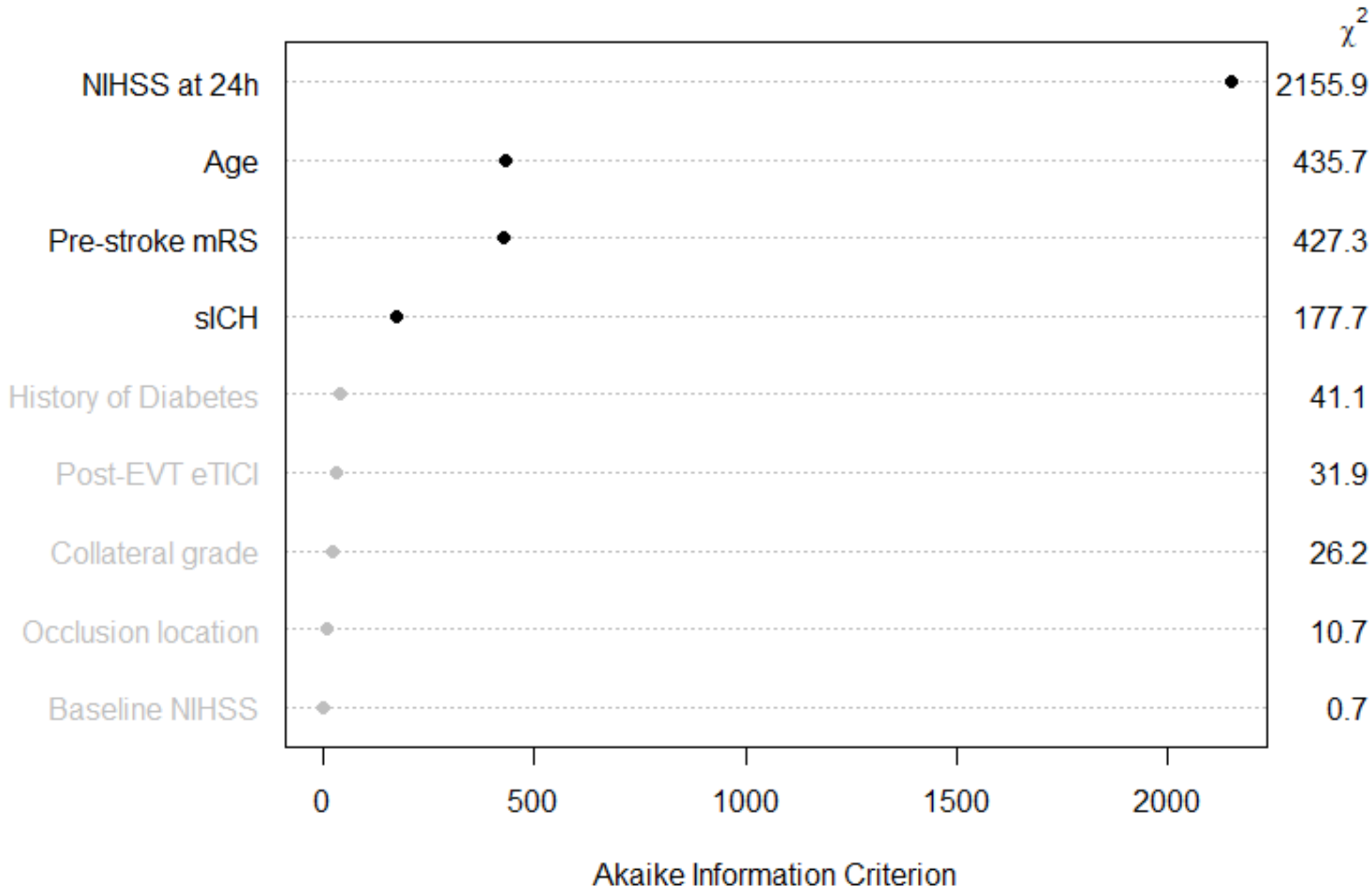

Note: Predictor selection is based on the Akaike information criterion (AIC) calculated from the pooled residual χ^2 statistic. Selected predictors are indicated with black circles. This approach helps balance model fit and complexity.

Abbreviations: mRS, modified Rankin Scale; eTICI, expanded treatment in cerebral infarction; NA, not applicable; NIHSS, National Institutes of Health Stroke Scale; sICH, symptomatic intracranial hemorrhage.
